# Solubility Modeling for Key Organic Compounds Used in Adavosertib (Anticancer API) Manufacturing

**DOI:** 10.1021/acsomega.5c01526

**Published:** 2025-06-25

**Authors:** Matthew Blair, Mazaher M. Chalchooghi, Robert J. Cox, Dimitrios I. Gerogiorgis

**Affiliations:** † Institute for Materials and Processes (IMP), School of Engineering, 3124 University of Edinburgh, The King’s Buildings, Edinburgh EH9 3FB, United Kingdom; ‡ Chemical Development, Pharmaceutical Technology and Development, Operations, 4978 AstraZeneca, Macclesfield SK10 2NA, United Kingdom

## Abstract

The solubility of organic compounds plays an important role in pharmaceutical manufacturing since key unit operations such as reactors, crystallizers, and solvent extraction units all rely on successful reagent and product dissolution. Consequently, predictive solubility modeling is very useful when developing new pharmaceutical processes, so as to minimize the experimentation required to understand the solubility of new molecular entities (NMEs). The present paper has thus employed the Non-Random Two-Liquid Segment Activity Coefficient (NRTL-SAC) model to describe the solubility of six organic compounds used in the production of an experimental anticancer drug, Adavosertib (specifically: AZD1775 Adavosertib Maleate, AZD1775 Aniline Maleate, AZD1775 Nitropip, AZD1775 Hydroxymethylsulfanyl, AZD1775 Bromopyridine.HBr, and AZD1775 Pyrimidine). The NRTL-SAC model has also been employed to estimate the melting temperature and enthalpy of fusion of these compounds, circumventing various difficulties arising in direct measurements (e.g., endothermic/exothermic phenomena near the melting point).

## Graphical abstract

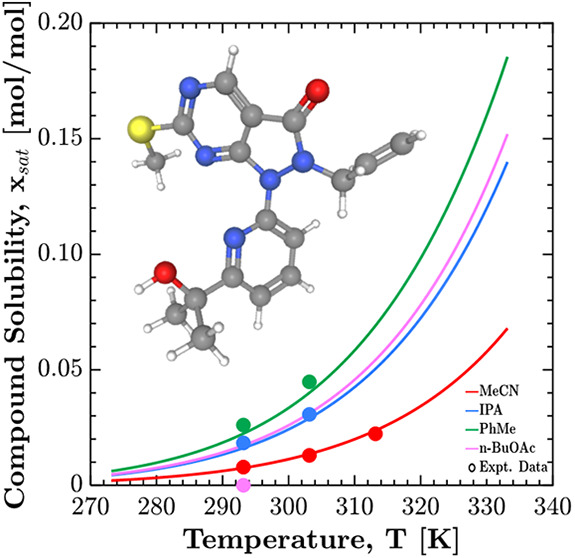

## 1 Introduction

The solubility of pharmaceutical compounds has an impact on the design of every unit operation used for their production since reactors, solvent extraction units, work-up procedures, and crystallization processes all depend on the successful dissolution of chemical species. Consequently, pharmaceutical companies are often forced to carry out extensive experimental campaigns aimed at ascertaining the behavior of new molecular entities (NMEs) in prospective solvents whenever they develop a new process. Solubility modeling can reliably and effectively help to reduce the size of these campaigns, thereby leading to substantial reductions in research and development (R&D) costs.

A multitude of relevant research studies have been conducted in recent years in order to model the solubility of pharmaceuticals (Table 1) using correlative, semi-predictive, or fully predictive computational approaches. The Apelblat^1–5^ and Non-Random Two-Liquid Activity Coefficient (NRTL) models^6,7^ emerge among the most popular correlative models available, whereas the Non-Random Two-Liquid Segment Activity Coefficient (NRTL-SAC) model^8–10^ (which is derived from the NRTL model) is the most widely used semi-predictive model. Beyond these, multiple predictive models are of course frequently used, e.g., the UNIQUAC Functional-group Activity Coefficient model (UNIFAC);^11–17^ its variants, the Modified UNIFAC (Dortmund)^18–28^ and Pharma UNIFAC^29^ models; the Perturbed Chain Statistical Associating Fluid Theory (PC-SAFT);^30,31^ the Conductor-like Screening Model for Real Solvents (COSMO-RS);^32–34^ and the COSMO Segment Activity Coefficient (COSMO-SAC)^35–37^ model (Table 2).

**Table 1:** Solubility Studies a Employed Computational Models for Select Pharmaceutical Compounds

	pharmacological classification	solubility model used	refs
aspirin	NSAID, antiplatelet	NRTL-SAC	^10^
camphor	topical antiseptic, analgesic antipruritic	NRTL-SAC	^10^
ephedrine	sympathomimetic	NRTL-SAC	^10^
lidocaine	local anesthetic, antiarrhythmic	NRTL-SAC	^10^
methylparaben	antifungal	NRTL-SAC	^10^
testosterone	hormone	NRTL-SAC	^10^
theophylline	bronchodilator	NRTL-SAC	^10^
estriol	hormone	NRTL-SAC	^10^
estrone	hormone	NRTL-SAC	^10^
morphine	narcotic analgesic	NRTL-SAC	^10^
piroxicam	NSAID	NRTL-SAC	^10^
hydrocortisone	corticosteroid	NRTL-SAC	^10^
haloperidol	typical antipsychotic	NRTL-SAC	^10^
risperidone	atypical antipsychotic	NRTL-SAC	^38^
fenofibrate	antilipemic agent	NRTL-SAC	^38^
tolbutamide	sulfonylurea	COSMO-RS	^39^
		PC-SAFT	^39^
butamben	anesthetic	NRTL-SAC	^38^
		Pharma UNIFAC	^38^
salicylamide	analgesic, antirheumatic	NRTL-SAC	^38^
artemisinin	antimalarial	UNIFAC	^40^
ibuprofen	NSAID	UNIFAC	^41^
		Pharma UNIFAC	^29^
		COSMO-SAC	^42^
		COSMO-RS	^39^
		PC-SAFT	^43^
diphenhydramine	antihistamine	UNIFAC	^44^
lovastatin	statin (HMG-CoA reductase inhibitor)	NRTL-SAC	^45^
		Apelblat	^2^
danazol	hormone	COSMO-SAC	^42^
isoniazid	antituberculosis agent	COSMO-SAC	^42^
flurbiprofen	NSAID	Apelblat	^1^
indomethacin	NSAID	COSMO-RS	^39^
		PC-SAFT	^39^
4-hydroxycoumarin	anticoagulant	COSMO-SAC	^42^
acetylcysteine	mucolytic agent	COSMO-SAC	^42^
coumarin	anti-inflammatory, anticoagulant, antimicrobial, anticonvulsant	COSMO-SAC	^42^
pyrazinamide	antituberculosis agent	COSMO-SAC	^42^
acyclovir	antiviral nucleoside analogue	COSMO-SAC	^42^
albendazole	anthelmintic	COSMO-SAC	^42^
paracetamol	analgesic, antipyretic	NRTL-SAC	^46^
		Pharma UNIFAC	^29^
		COSMO-SAC	^42^
		COSMO-RS	^39^
		PC-SAFT	^39,43,46^
naproxen	NSAID	NRTL-SAC	^46^
		COSMO-RS	^39^
		PC-SAFT	^46^
sulfadiazine	antibiotic	NRTL-SAC	^45^
		PC-SAFT	^43^
salicylic acid	keratolytic agent	NRTL-SAC	^46^
		PC-SAFT	^46^
temazepam	benzodiazepine	NRTL-SAC	^9,46^
		PC-SAFT	^46^
carbamazepine	anticonvulsant	COSMO-RS	^39^
		PC-SAFT	^39^
griseofulvin	antifungal	COSMO-RS	^39^
		PC-SAFT	^39^
ribavirin	antiviral nucleoside analogue	COSMO-RS	^39^
		PC-SAFT	^39^
valsartan	angiotensin receptor blocker	COSMO-RS	^39^
		PC-SAFT	^39^

**Table 2:** Key Attributes of Published Solubility Models

name/acronym	model nature	complexityComplexity	refs
Apelblat	correlative	low	^1–5^
NRTL	correlative	low–medium	^6,7,47^
NRTL-SAC	semipredictive	low–medium	^9,10,29,48^
UNIFAC	predictive	medium	^11–17,29^
modified UNIFAC (Dortmund)	predictive	medium	^18–29^
Pharma UNIFAC	predictive	medium	^29^
PC-SAFT	predictive	high	^30,31,39,47^
COSMO-RS	predictive	high	^29,32–34,47^
COSMO-SAC	predictive	high	^35–37,48^

Nevertheless, it can be difficult to identify which of these approaches and models will best suit a given application, since very few studies actually compare all of these models against one another. The majority of published studies report their results using metrics which depend upon the size of the experimental data sets that they use, e.g., mean square error (MSE) and root-mean-square log error (RMSLE), making it difficult to make fair comparisons with other studies. Semi-predictive and predictive models are however more advantageous than correlative models. Careful comparisons across different studies yield meaningful conclusions about these models specifically.

An example of how this can be performed is presented in Table 3, where each of the semi-predictive and predictive models has been compared for three active pharmaceutical ingredients (APIs): ibuprofen, paracetamol, and butamben. Therein, one clearly observes that the NRTL-SAC model most often outperforms the other models considered, often reporting lower MSE and RMSLE values when applied to identical experimental data sets (represented in Table 3 by cells of the same color).

**Table 3:**
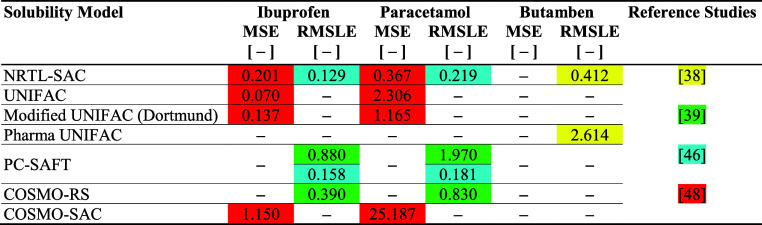
Comparison of Semi-Predictive and Predictive Solubility Models for Specific APIs in Multiple Literature Studies

These findings echo results published by several authors in recent years, which have compared a subset of the models presented in Table 3. For instance, Klajmon^39^ recently compared PC-SAFT and COSMO-RS for a variety of pharmaceutical compounds, ultimately concluding that COSMO-RS outperformed PC-SAFT for the drugs studied (e.g., carbamazepine, ibuprofen, paracetamol). Moreover, Diedrichs and Gmehling^29^ systematically compared the UNIFAC, Modified UNIFAC (Dortmund), Pharma UNIFAC, COSMO-RS, and NRTL-SAC models for a variety of active pharmaceutical ingredients (APIs) and concluded that the NRTL-SAC model provided the best results. Bouillot et al.^48^ have bolstered these findings in their study examining the UNIFAC, Modified UNIFAC (Dortmund), COSMO-SAC, and NRTL-SAC models: they conclude that UNIFAC provides more accurate estimations than COSMO-SAC, while also noting that both UNIFAC and NRTL-SAC can be used effectively to guide experimental campaigns (minimizing their size and cost as a result). What is more, several studies note the ability of NRTL-SAC to accurately predict solubility trends for pharmaceuticals in pure and mixed solvents when parameterized from extremely small experimental data sets.^8–10,38,45,49^ This is extremely valuable during the early stages of drug development, when only limited amounts of pharmaceutical materials (especially products) are available for laboratory testing.

Accordingly, the NRTL-SAC model can be identified as the best (and most practical) way to model the solubility of organic compounds during the early stages of drug development. However, it should be acknowledged that this model does not always promise quantitative results^8–10,50^ and rather offers reasonable approximations of any trends likely to occur. Consequently, in the present paper, the NRTL-SAC activity coefficient model has been used to study the solubility of six organic compounds (Figure 1) required for the manufacture of an experimental anticancer drug, Adavosertib.

**Figure 1:**
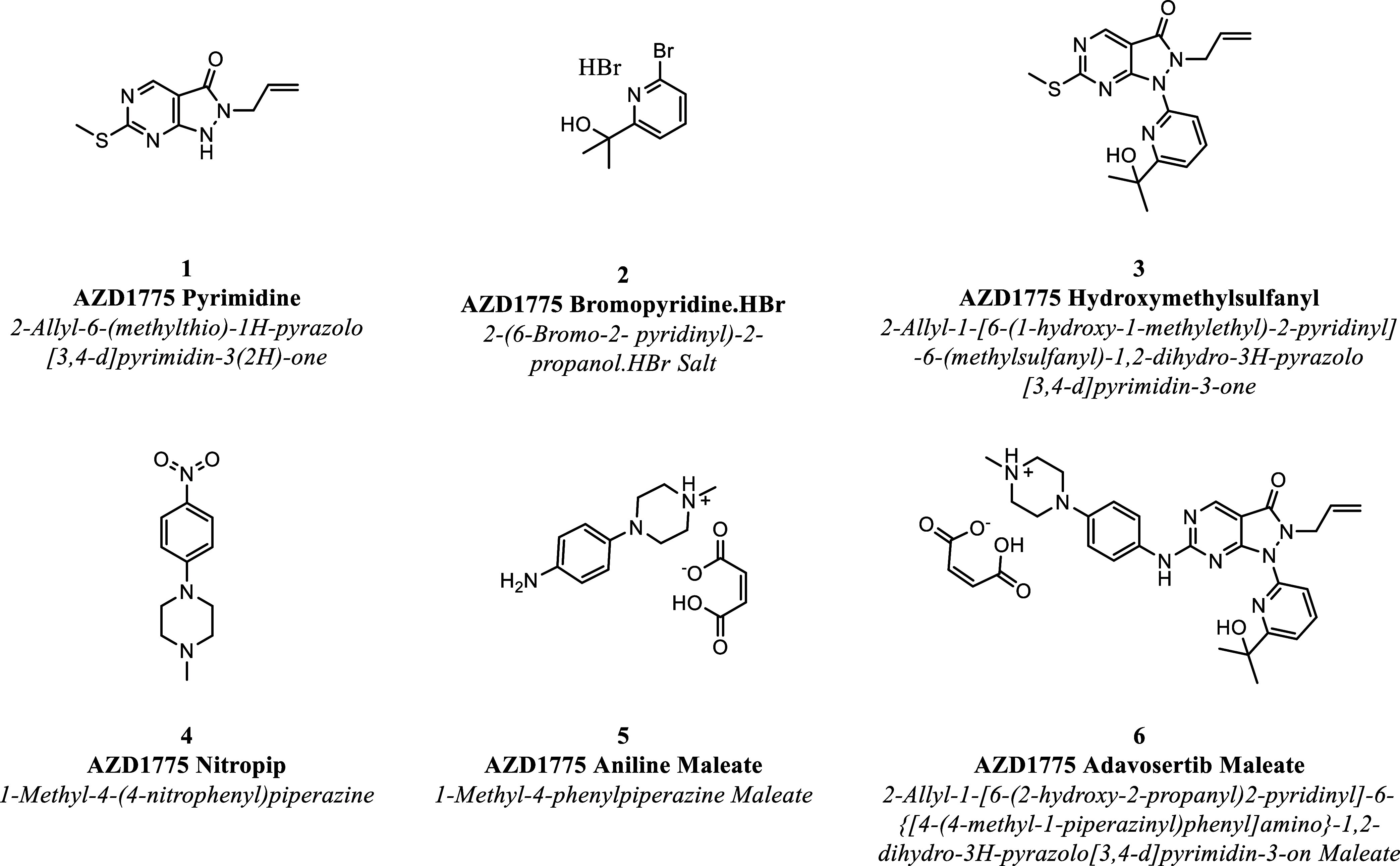
Chemical structures and full (IUPAC-standard) names of all organic compounds studied.

Remarkably, the simple structure of the NRTL-SAC model makes it computationally inexpensive.^38,51^ Thus, the methodology of the present contribution can also be used in the future to identify cost-optimal and environmentally friendly manufacturing routes towards each of the compounds in Figure 1,^40,41,52–56^ hence consequently incorporated within optimization architectures to compare different process flowsheets for drug production.^44,52,57–59^ Published studies have already successfully demonstrated how promising crystallization processes can be identified by efficiently using solubility models to screen hundreds of solvent candidates before selecting the one that allows the highest crystal yield, smallest carbon footprint, or lowest process costs.^45,59,60^ Process Systems Engineering (PSE) methodologies can be incredibly useful towards drastically reducing the amount of solvent waste currently sent to sewers by pharmaceutical companies across major markets (Figure 2),^61^ given the growing waste disposal costs of the global pharmaceutical sector (∼30 billion USD p.a. worldwide^62,63^ ).

**Figure 2:**
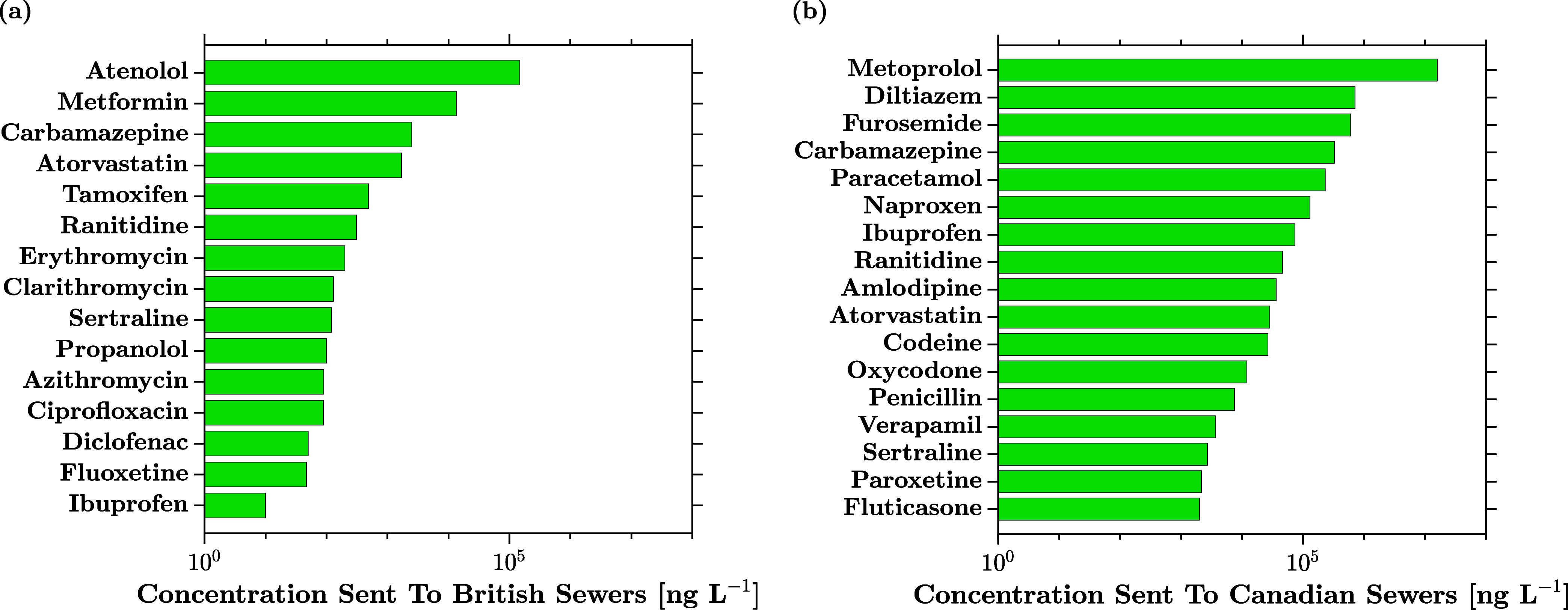
API concentrations in (a) British and (b) Canadian sewers due to effluent discharge from pharmaceutical manufacturing sites (Data: ref 61).

## 2 Principles of Solubility Modeling

To conduct this computational analysis, the solubility of each organic compound has been modeled using eq 1, which, upon expansion affords eq 2. The latter can then be simplified to yield eq 3, because sensible heating effects of the latter two terms of eq 2 are considered negligible.

1 $$\ln a_{\mathrm{solid}}=\ln x_{\mathrm{sat}}+\ln\gamma_{\mathrm{sat}}$$

2 $$\ln a_{\mathrm{solid}}=\frac{\Delta H_{\mathrm{fusion}}}{R}\left(\frac{1}{T_{\mathrm{mp}}}-\frac{1}{T}\right)+\frac{1}{R}\int_{T_{\mathrm{mp}}}^{T}\frac{\Delta C_{p}}{T}dT-\frac{1}{\mathrm{RT}}\int_{T_{\mathrm{mp}}}^{T}\Delta C_{p}dT$$

3 $$\ln x_{\mathrm{sat}}=\frac{\Delta H_{\mathrm{fusion}}}{R}\left(\frac{1}{T_{\mathrm{mp}}}-\frac{1}{T}\right)-\ln\gamma_{\mathrm{sat}}$$

Consequently, the solubility of each compound can be estimated by using the NRTL-SAC model to compute the activity coefficient (γ_sat_) if the NRTL-SAC parameters (*r*_*X*_, *r*_*Y* ^–^_, *r*_*Y* ^+^_, *r*_*Z*_), enthalpy of fusion (Δ*H*_fusion_) and melting temperature (*T*_mp_) associated with each species are known. However, if these parameters are not known, then experimental solubility data should be used to estimate their numerical values by fitting eq 3 using reliable parameter estimation methods.

The NRTL-SAC model has thus been used in this study to describe the activity coefficient of the different compounds portrayed in Figure 1 by fitting experimental solubility data collected across a range of solvents (Table 4) and temperatures (293.15, 303.15, and 313.15 K) to eq 3. The key contribution is thus the development of a semi-predictive model which can also be used in the future to predict the solubility of each compound in solvents for which experimental data is entirely unavailable.

**Table 4:** Thermophysical Properties of Compounds Used in This Study^a^

species	name	CAS no.	chemical formula	*M* [g mol^–1^]	*T*_bp_ [K]	*T*_mp_ [K]	refs
1	AZD1775 pyrimidine	955368-90-8	C_9_H_10_N_4_OS	222.27	671.05		^64,65^
2	AZD1775 bromopyridine HBr		C_8_H_11_Br_2_NO	296.99			
3	AZD1775 hydroxymethylsulfanyl	955369-56-9	C_17_H_19_N_5_O_2_S	357.43	838.75	374.15*	^64,65^
4	AZD1775 nitropip	16155-03-6	C_11_H_15_N_3_O_2_	221.26	642.65	377.15*	^64,65^
5	AZD1775 aniline maleate		C_15_H_21_N_3_O_4_	307.35		390.15*	
6	AZD1775 adavosertib maleate		C_31_H_36_N_8_O_6_	616.68		445.15*	
MeCN	acetonitrile	75-05-8	C_2_H_3_N	41.05	354.82	228.15	^65^
IPA	isopropyl alcohol	67-63-0	C_3_H_8_O	60.10	355.93	184.82	^65^
*n*-BuOAc	*n*-butyl acetate	123-86-4	C_6_H_12_O_2_	116.16	398.71	195.93	^65^
PhMe	toluene	108-88-3	C_7_H_8_	92.14	384.26	178.15	^65^

a *: our own estimation

The methodology used to conduct this analysis is discussed in Section 3. Thermophysical properties of each of the solvents and pharmaceutical compounds considered in our computations (with certain original estimations) are provided in Table 4.

## 3 Parameterization of the NRTL-SAC Model

The following section outlines the principles of the NRTL-SAC Equation of State (EoS), and the parameter estimation procedure used for its parameterization in this study.

### 3.1 The NRTL-SAC Model

The NRTL-SAC EoS^8–10^ expresses the activity coefficient of chemical species using combinatorial and residual activity coefficients, as shown in eq 4. To do this, it uses four conceptual segments to describe the interactions among different molecules. Namely, a hydrophilic segment (*X*) representing polar molecular surfaces capable of forming a hydrogen bond; a hydrophobic segment (*Z*) covering areas that resist the formation of hydrogen bonds; and two polar segments (*Y* ^+^ and *Y* ^–^) that account for the fact that molecules can have attractive (electron-pair donor site) and/or repulsive (electron-pair acceptor site) interactions with one another.^10^

4 $$\ln\gamma_{I}=\ln\gamma_{I}^{R}+\ln\gamma_{I}^{C}$$

The full set of equations used by the NRTL-SAC model to achieve this is presented in eqs 5–13 below. Residual activity coefficients can be calculated using eqs 5–10 alone, while combinatorial coefficients can be determined using the Flory–Huggins term presented in eqs 11–13.

5 $$\ln\gamma_{I}^{R}=\sum_{m}r_{m,I}\left[\ln\Gamma_{m}^{\mathrm{lc}}-\ln\Gamma_{m}^{\mathrm{lc},I}\right]$$

6 $$\ln\Gamma_{m}^{\mathrm{lc}}=\frac{\sum_{j}x_{j}G_{\mathrm{jm}}\tau_{\mathrm{jm}}}{\sum_{k}x_{k}G_{\mathrm{km}}}+\sum_{m^{\prime }}\frac{x_{m\prime }G_{\mathrm{mm}\prime }}{\sum_{k}x_{k}G_{\mathrm{km}\prime }}\left(\tau_{\mathrm{mm}\prime }-\frac{\sum_{j}x_{j}G_{\mathrm{jm}\prime }\tau_{\mathrm{jm}\prime }}{\sum_{k}x_{k}G_{\mathrm{km}\prime }}\right)$$

7 $$\ln\Gamma_{m}^{\mathrm{lc},I}=\frac{\sum_{j}x_{j,I}G_{\mathrm{jm}}\tau_{\mathrm{jm}}}{\sum_{k}x_{k,I}G_{\mathrm{km}}}+\sum_{m^{\prime }}\frac{x_{m\prime ,I}G_{\mathrm{mm}\prime }}{\sum_{k}x_{k,I}G_{\mathrm{km}\prime }}\left(\tau_{\mathrm{mm}\prime }-\frac{\sum_{j}x_{j,I}G_{\mathrm{jm}\prime }\tau_{\mathrm{jm}\prime }}{\sum_{k}x_{k,I}G_{\mathrm{km}\prime }}\right)$$

8 $$G_{\mathrm{ij}}=\exp\left(-\alpha_{\mathrm{ij}}\tau_{\mathrm{ij}}\right)$$

9 $$x_{j}=\frac{\sum_{J}x_{J}r_{j,J}}{\sum_{I}\sum_{i}x_{I}r_{i,I}}$$

10 $$x_{j,I}=\frac{r_{j,I}}{\sum_{i}r_{i,I}}$$

11 $$\ln\gamma_{I}^{C}=1+\ln\left(\frac{\varphi_{I}}{x_{I}}\right)-r_{I}\sum_{J}\frac{\varphi_{J}}{r_{J}}$$

12 $$r_{I}=\sum_{i}r_{i,I}$$

13 $$\varphi_{I}=\frac{r_{I}x_{I}}{\sum_{J}r_{J}x_{J}}$$

The binary interaction parameter values required in order to implement the NRTL-SAC model are listed in Table 5.

**Table 5:** Temperature-Independent Binary Interaction Parameter Values Used for Each Conceptual Segment–Segment Interaction Allowed by the NRTL-SAC Model^9,10^

segment 1	*X*	X	*Y* ^–^	*Y* ^+^	*X*
segment 2	*Y* ^–^	*Z*	*Z*	*Z*	*Y* ^+^
τ_12_	1.643	6.547	–2.000	2.000	1.643
τ_21_	1.834	10.949	1.787	1.787	1.834
α_12_ = α_21_	0.200	0.200	0.300	0.300	0.200

### 3.2 Parameter Estimation Methodology

As a consequence of the nonlinear nature of the solubility equations described in Section 2 and the NRTL-SAC model itself, a multistart parameter estimation procedure has been developed in MATLAB to parameterize the NRTL-SAC model as described in Section 2. A summary of this methodology is provided in the flowchart depicted by Figure 3; the code thus employs MATLAB’s *fminsearch* tool to minimize the value of the objective function defined by eqs 14 and 15 when run from 1000 different start-points. Importantly, the code constrains the values of each parameter estimated to fall within reasonable (user-specified) bounds (Table 6), thereby ensuring that only parameters with physical meaning are obtained.

**Figure 3:**
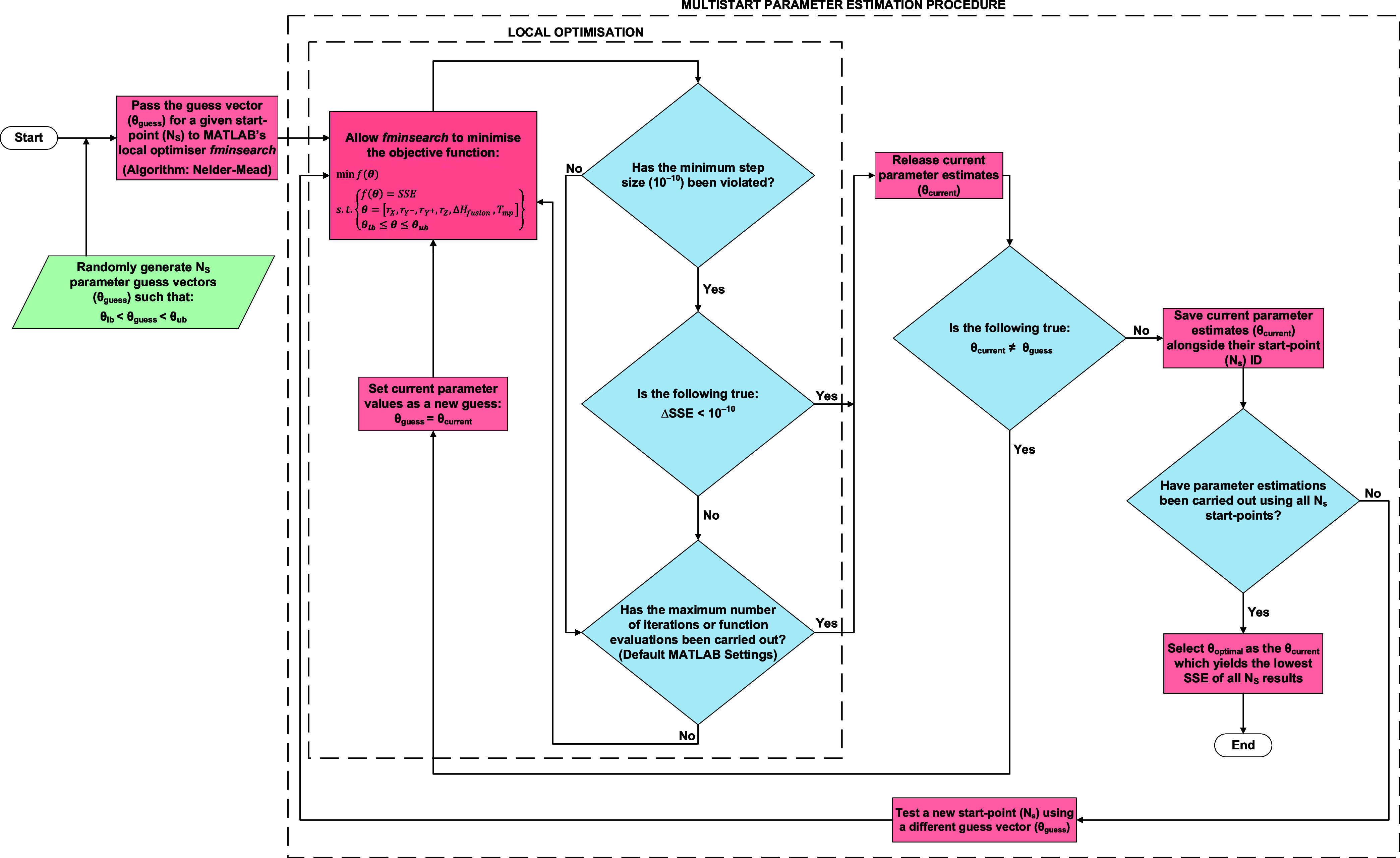
The multistart parameter estimation procedure hereby employed for solubility modeling.

**Table 6:** Parameter Bounds for NRTL-SAC Modeling

	*r*_*X*_	*r*_*Y* ^–^_	*r*_*Y* ^+^_	*r*_*Z*_	Δ*H*_fusion_ [kJ mol^–1^]	*T*_mp_ [K]
lower bound (θ_lb_)	0	0	0	0	1	273.15
upper bound (θ_ub_)	5	5	5	5	1000	500.00

Accordingly, it has been stipulated that any NRTL-SAC parameters (*r*_*m*,*I*_) for each solute must be real and greater than (or equal to) zero, but less than 5. The reason is that molecules cannot have a negative number of molecular segments. Pharmaceuticals previously studied using the NRTL-SAC model have exhibited *r*_*m*,*I*_ values between 1 and 2.5, while having molecular masses roughly half that of the largest compound investigated here.^10,38^ Moreover, enthalpies of fusion here must be real values between 1–1000 kJ mol^–1^ since the vast majority of pharmaceuticals exhibit enthalpies of fusion of between 10 and 100 kJ mol^–1^,^10,38–44,46,58,59,66^ although some values may fall outwith this range for certain substances.

14 $$\mathrm{SSE}=\frac{1}{2}\sum_{n=1}^{N_{\mathrm{temp}}}\sum_{j=1}^{N_{\mathrm{solvents}}}{\left(x_{I}^{\mathrm{expt}}-x_{I}^{\mathrm{model}}\right)}^{2}$$

15 $$\begin{array}{l} \min f\left(\theta \right)\\ s.t.\;\left\{\begin{array}{l} f\left(\theta \right)=\mathrm{SSE}\\ \theta =\left[r_{X},r_{Y^{-}},r_{Y^{+}},r_{Z},\Delta H_{\mathrm{fusion}},T_{\mathrm{mp}}\right]\\ \theta_{\mathrm{lb}}\leq \theta \leq \theta_{\mathrm{ub}} \end{array}\right\} \end{array}$$

To enable reliable computations, four solvents have been considered to provide the experimental solubility data required to correlate each solute with the NRTL-SAC model. Specifically, acetonitrile (MeCN), isopropyl alcohol (IPA), *n*-butyl acetate (*n*-BuOAc), and toluene (PhMe) together cover the full range of polar, hydrophobic, and hydrophilic segments likely to be found in the pharmaceutical compounds tested,^10,38^ thereby aligning with the minimum data requirements prescribed by Chen and Song in their original NRTL-SAC model development.^8–10^

Importantly, the solubility of each compound in these four solvents has been measured over a wide range of temperatures (293.15–313.15 K) to allow better estimations of the melting temperatures and enthalpies of fusion associated with each compound. The need to computationally estimate the melting temperature of a compound only emerges when the latter cannot be determined experimentally via Differential Scanning Calorimetry (DSC). Here, it was only necessary to estimate the melting temperature of compounds **1**–**2** since exothermic and endothermic activity was observed immediately on either side of the melting point for these substances.

Following successful parameter estimation, the root-mean-square log error, RMSLE (eq 16) h also been calculated for each solute, allowing our original results to be compared with those reported by similar published studies.

16 $$\mathrm{RMSLE}={\left[\sum_{n=1}^{N_{\mathrm{temp}}}\sum_{j=1}^{N_{\mathrm{solvents}}}\frac{1}{N_{\mathrm{data}}}{\left(\ln x_{I}^{\mathrm{expt}}-\ln x_{I}^{\mathrm{model}}\right)}^{2}\right]}^{1/2}$$

## 4 Discussion of Results

### 4.1 Solubility Predictions

NRTL-SAC parameters calculated for each compound using the methods discussed in Section 3 are presented in Table 7. The melting temperature, enthalpy of fusion, and molecular segment values estimated for each solute match typical values reported for small molecules in the literature, with all six compounds exhibiting enthalpies of fusion in the range of 10–100 kJ mol^–1^,^10,38–44,46,58,59,66^ at low RMSLE values of 0.01–10.^8–10,29,38,46,48^

**Table 7:** NRTL-SAC Solubility Model Parameter Estimation Results

solute	name	*r*_*X*_	*r*_*Y* ^–^_	*r*_*Y* ^+^_	*r*_*z*_	Δ*H*_fusion_ [kJ mol^–1^]	*T*_mp_ [°K]	SSE [(mol/mol)^2^]	RMSLE
1	AZD1775 pyrimidine	0.000	0.000	0.000	3.477	34.920	405.40	5.461 × 10^–7^	3.067
2	AZD1775 bromopyridine HBr	0.000	0.101	0.000	2.757	12.393	442.21	6.466 × 10^–6^	4.206
3	AZD1775 hydroxymethylsulfanyl	0.488	0.076	0.000	0.000	43.151		1.704 × 10^–4^	6.245 (0.096)^a^
4	AZD1775 nitropip	0.761	0.000	0.000	0.000	29.249		6.538 × 10^–4^	0.417
5	AZD1775 aniline maleate	0.000	0.076	0.336	0.439	59.461		8.027 × 10^–6^	0.858
6	AZD1775 adavosertib maleate	0.000	0.000	0.856	2.620	64.810		9.652 × 10^–9^	4.447

a Bracketed RMSLE values exclude *n*-BuOAc solubilities.

The solubility profiles predicted by NRTL-SAC have been plotted against experimental data for each compound (Figure 4). Trends in compound solubility are thus successfully captured by the NRTL-SAC model, with the exception of compound **3** in *n*-BuOAc (Figure 4c). The significant underlying root cause for this is the difficulty to measure the solubility of compound **3** in *n*-BuOAc due to its persistent degradation tendency, hence we conclude that *n*-BuOAc can be excluded from the formal analysis for compound **3** (cf. adjusted RMSLE, Table 7).

**Figure 4:**
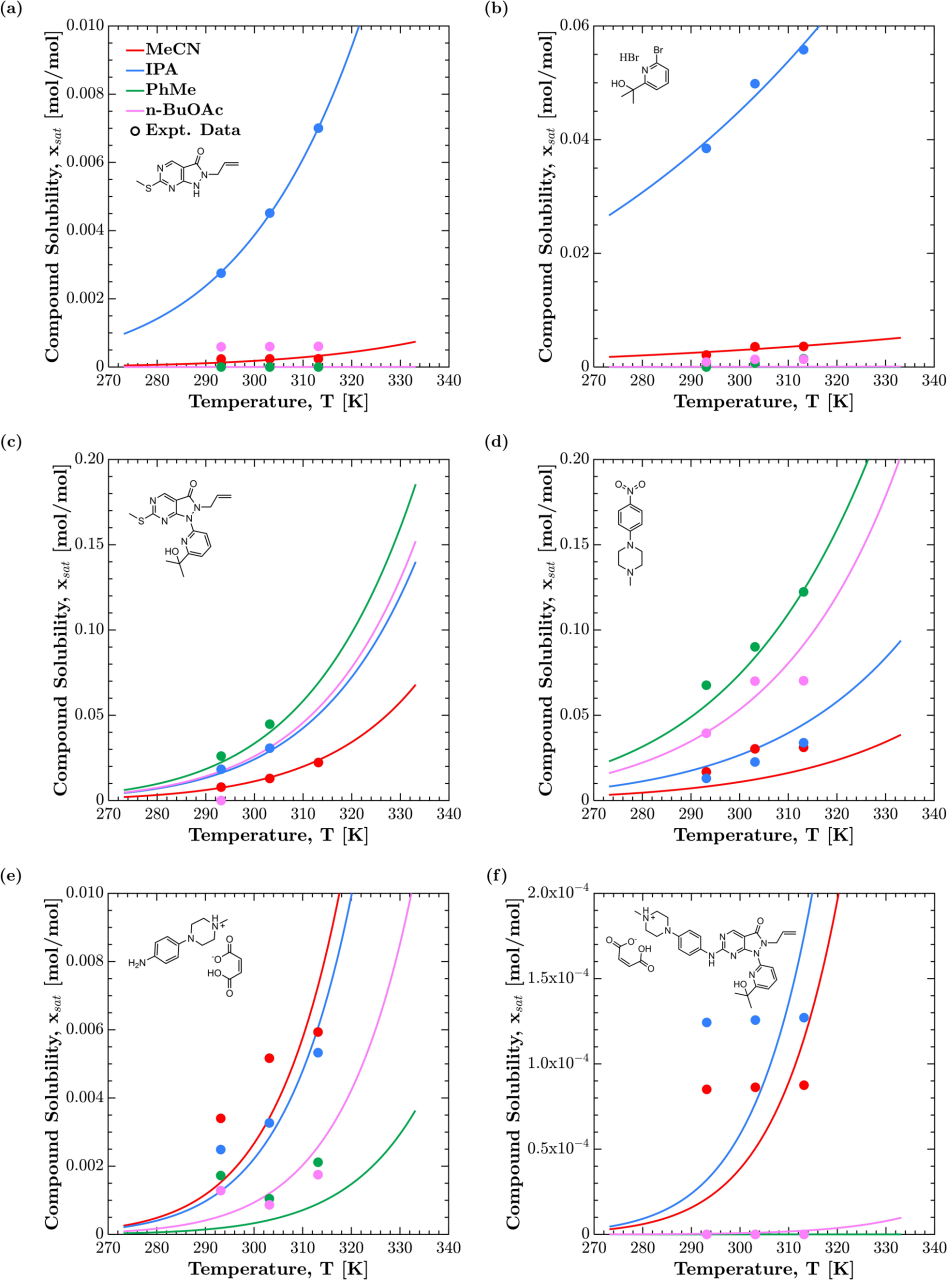
Solubility data and model curves for all solvent–solute combinations: (a) AZD1775 Pyrimidine, (b) AZD1775 Bromopyridine.HBr, (c) AZD1775 Hydroxymethylsulfanyl, (d) AZD1775 Nitropip, (e) AZD1775 Aniline Maleate, and (f) AZD1775 Adavosertib Maleate.

Due to these observations, future NRTL-SAC applications should assume that compound **3** will degrade in *n*-BuOAc or similar solvents (unless compound reactivity is to be explicitly addressed). Nevertheless, we conclude that (for nonreacting systems) the NRTL-SAC model can correctly predict:

(1) The magnitude of a solute’s solubility in different solvents
(2) The relative solubility of different solutes in common solvents
(3) The rough temperature dependence associated with a solute’s solubility

To bolster these conclusions, Figure 5 demonstrates the ability of NRTL-SAC to predict the relative magnitude of a solute’s solubility in different solvents, showing that predicted solubilities closely match experimental observations for the compounds studied in this study.bFurthermore, Figure 5 also demonstrates that the NRTL-SAC model retains its predictive capabilitybfor compounds with extremely low solubilities (e.g., compound **6**, whose solubility is a full order of magnitude smaller than the other five compounds). Even in case of data scarcity, this can greatly benefit the search for candidate solvents for new pharmaceutical manufacturing processes.

**Figure 5:**
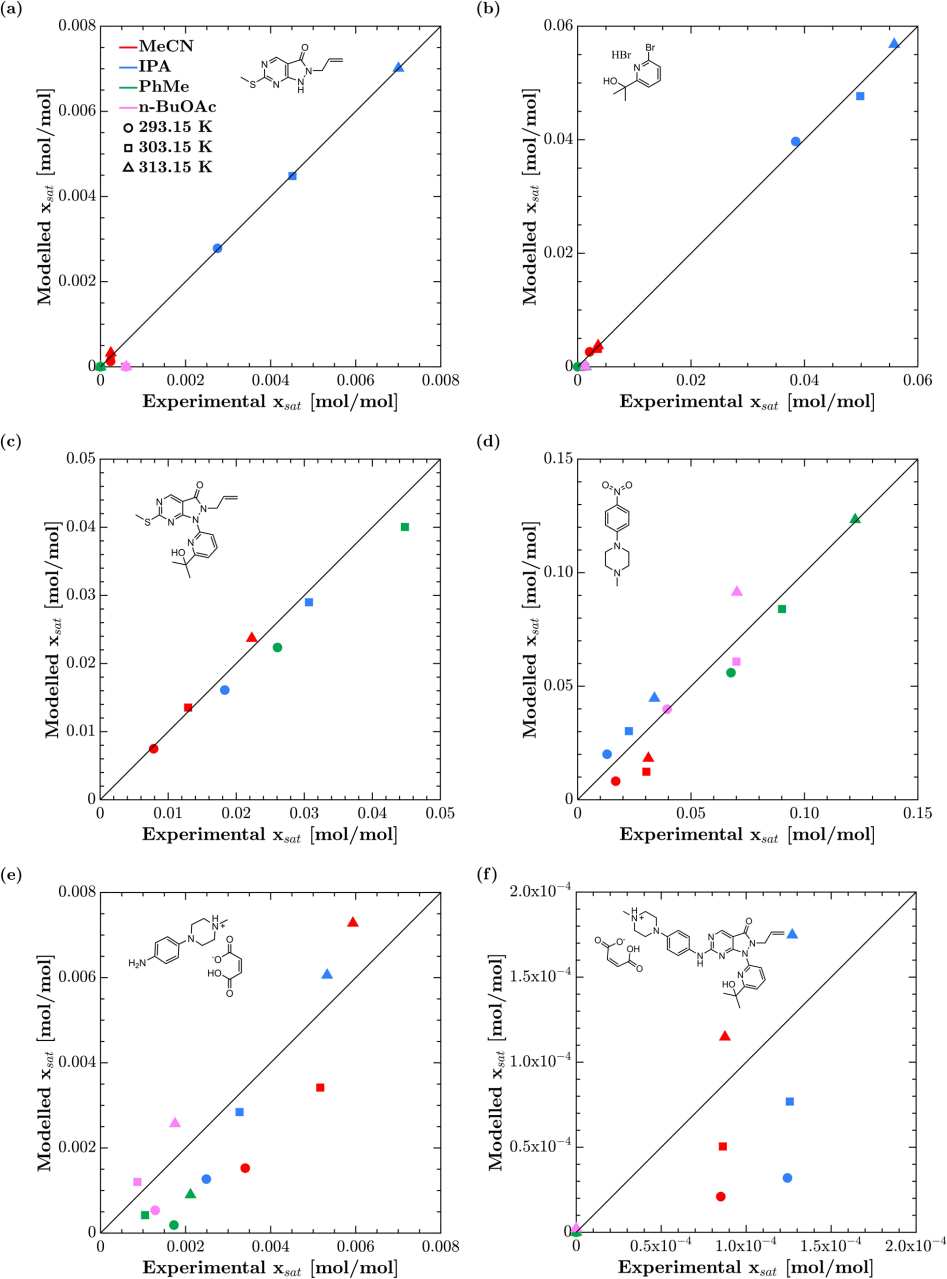
Parity plots of modeled vs experimental solubilities for four solvents and three temperatures: (a) AZD1775 Pyrimidine, (b) AZD1775 Bromopyridine.HBr, (c) AZD1775 Hydroxymethylsulfanyl, (d) AZD1775 Nitropip, (e) AZD1775 Aniline Maleate and (f) AZD1775 Adavosertib Maleate.

### 4.2 Performance of Multistart Parameter Estimation

Figure 6 highlights the nontrivial nature of the EoS parameterization problem examined in this application, demonstrating the importance of using multiple start-point methods to solve nonlinear parameter estimation problems. Specifically, a large proportion of initial guesses supplied to the optimization solver results in calculating suboptimal model parameters due to local minima. Banding is also observed (due to imposed bounds), with several solutions within.

**Figure 6:**
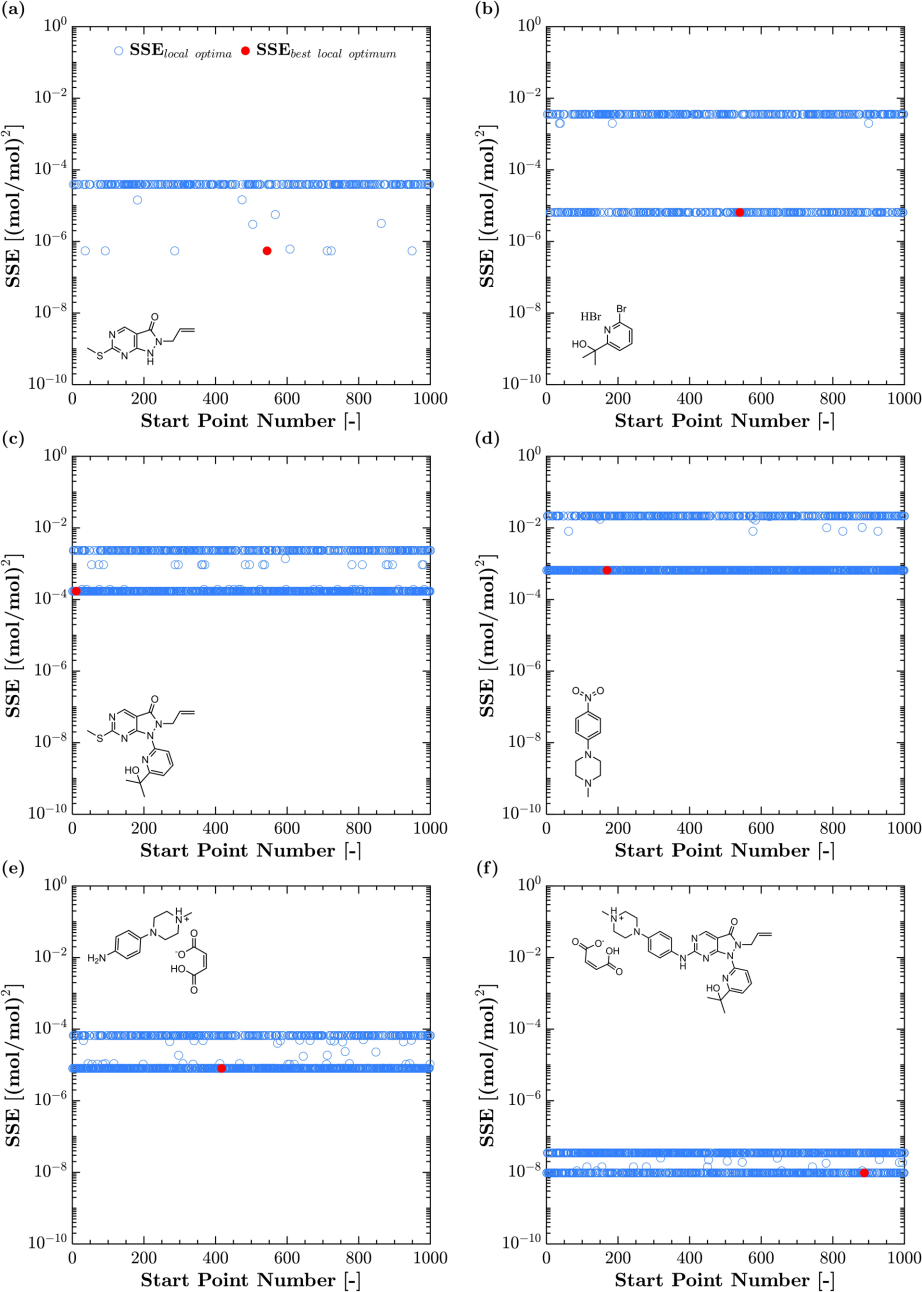
Objective function values for all 1000 start-points in parameter estimation: (a) AZD1775 Pyrimidine, (b) AZD1775 Bromopyridine.HBr, (c) AZD1775 Hydroxymethylsulfanyl, (d) AZD1775 Nitropip, (e) AZD1775 Aniline Maleate, and (f) AZD1775 Adavosertib Maleate.

## 5 Conclusions

The present study illustrates how the NRTL-SAC activity coefficient model has been successfully parameterized for six pharmaceutical compounds (Figure 1) required for the production of the experimental anticancer drug, Adavosertib, thereby demonstrating the viability of this methodological option during early-stage drug development. Moreover, it paves the way for future process modeling studies aimed at identifying cost-optimal and environmentally friendly manufacturing routes for Adavosertib, because reliable solubility prediction is a prerequisite for modelling the separation processes (e.g., liquid extractions, crystallizations) required for its production.

In general, we observe that NRTL-SAC model parameters reported for complex and novel compounds may not achieve fully quantitative predictions in case of data scarcity. Nevertheless, they offer qualitative descriptions as to how each compound is likely to behave in different solvents (hence capture the relative solubility of each compound in different solvents, while describing their rough temperature dependence).

Consequently, the results presented herein should be used to guide subsequent experimental campaigns rather than replacing them entirely, helping to cut down on the number of experimental trials needed by offering insight into the likely behavior of each compound in untested solvent mixtures. This will be extremely useful in order to identify the best solvent (or solvent mixture) available for any given step in Adavosertib production. Moreover, we remark that the NRTL-SAC model cannot predict the reactivity (or possible degradation) of a solute in a given solvent (as mentioned in Section 4). Thus, careful consideration is required for solvent candidate selection in these studies, to eliminate reaction phenomena.

## 6 Future Work

Future work should include validation of the model parameters presented herein: comparing any predictions for as-yet untested solute–solvent combinations with new experimental observations. Ultimately, the ability to predict trends for new systems (either quantitatively or qualitatively) has great importance, but also implies additional experimental effort.

To perform further studies, the solubility of each compound would need to be measured in an array of new solvents at a range of process temperatures, as it is of interest to see how well these parameters predict:

(1) Each solute’s solubility in binary water-organic mixtures (given water’s extensive use as a solvent and antisolvent within the pharmaceutical industry),
(2) Solute solubility near the operational limits of different solvents (i.e., near their melting and boiling points).

## Notes

Tabulated, plotted, and cited data presented in this study suffice for the reproduction of all parameter estimation results, and no other supporting data are required to ensure reproducibility.

## Nomenclature

**Latin Letters**
*a*_solid_: activity of a compound in its solid state
Δ*C*_p_: solid supercooled melt specific heat capacity difference [kJ mol^–1^ K^–1^]
*f*(**θ**): parameter estimation objective function
*G*_*ij*_: NRTL-SAC local binary quantity for interacting segments *i* and *j*
Δ*H*_fusion_: enthalpy of fusion [kJ mol^–1^]
M: molecular weight [g mol^–1^]
*N*_data_: number of data points
*N*_temp_: number of temperatures
*N*_S_: number of start-points initiated in the parameter estimation problem
*N*_solvents_: number of solvents tested
*r*_*I*_: NRTL-SAC total segment number for compound *I*
*r*_*m*,*I*_: number of NRTL-SAC segment *m* contained within compound *I*
R: universal gas constant [J mol^–1^ K^–1^]
T: system temperature [K]
*T*_bp_: boiling point temperature [K]
*T*_mp_: melting point temperature [K]
*x*_*j*_: NRTL-SAC segment-based mole fraction of segment *j* [mol mol^–1^]
*x*_*j*,*I*_: NRTL-SAC mole fraction of segment *j* in compound *I* [mol mol^–1^]
*x*_*I*_: NRTL-SAC mole fraction of compound *I* [mol mol^–1^]
*x*_*I*_^expt^: experimental mole fraction of compound *I* [mol mol^–1^]
*x*_*I*_^model^: modeled mole fraction of compound *I* [mol mol^–1^]
*x*_sat_: saturation solubility [mol mol^–1^]
X: NRTL-SAC hydrophobic segment
*Y* ^–^: NRTL-SAC negative polar segment
*Y* ^+^: NRTL-SAC positive polar segment
Z: NRTL-SAC hydrophilic segment
**Greek Letters**
α_*ij*_: NRTL-SAC nonrandomness factor between segments *i* and *j*
γ_*I*_^*C*^: NRTL-SAC combinatorial activity coefficient for compound *I*
γ_*I*_^*R*^: NRTL-SAC residual activity coefficient for compound *I*
γ_*I*_: NRTL-SAC overall activity coefficient for compound *I*
γ_sat_: saturation activity coefficient
Γ_*m*_^*lc*^: NRTL-SAC activity coefficient of segment species *m*
Γ_*m*_^*lc*,*I*^: NRTL-SAC activity coefficient of segment *m* within compound *I*
**θ**: parameter vector
**θ**_**lb**_: parameter vector lower bound
**θ**_**ub**_: parameter vector upper bound
τ_*ij*_: NRTL-SAC binary interaction parameter between segments *i* and *j*
φ_*I*_: NRTL-SAC segment mole fraction of compound *I*
**Acronyms**
RMSLE: root-mean-square log error
SSE: sum of square errors
**NRTL-SAC Subscripts**
*i*,*j*,*k*,*m*,*m*′: segment species indexes
*I*,*J*: chemical species indexes
